# Force Trends and Pulsatility for Catheter Contact Identification in Intracardiac Electrograms during Arrhythmia Ablation

**DOI:** 10.3390/s18051399

**Published:** 2018-05-02

**Authors:** David Rivas-Lalaleo, Sergio Muñoz-Romero, Mónica Huerta, Mayra Erazo-Rodas, Juan José Sánchez-Muñoz, José Luis Rojo-Álvarez, Arcadi García-Alberola

**Affiliations:** 1 Departamento de Eléctrica y Electrónica, Universidad de las Fuerzas Armadas ESPE, Av. General Rumiñahui s/n, 170501 Sangolquí, Ecuador; mjerazo@espe.edu.ec; 2 Department of Signal Theory and Communications and Telematic Systems and Computation, Rey Juan Carlos University, Fuenlabrada, 28943 Madrid, Spain; sergio.munoz@urjc.es (S.M.-R.); joseluis.rojo@urjc.es (J.L.R.-Á); 3 Center for Computational Simulation, Universidad Politécnica de Madrid; Boadilla, 28223 Madrid, Spain; 4 Universidad Politécnica Salesiana, 010105 Cuenca, Ecuador; mhuerta@ups.edu.ec; 5 Arrhythmia Unit, University Clinic Hospital Virgen de la Arrixaca, El Palmar, 30120 Murcia, Spain; juanjosanchezmunoz@me.com (J.J.S.-M.); arcadi@secardiologia.es (A.G.-A.)

**Keywords:** arrhythmias, Principal Component Analysis, linear classifiers, tissue-catheter contact, force signals, electrograms

## Abstract

The intracardiac electrical activation maps are commonly used as a guide in the ablation of cardiac arrhythmias. The use of catheters with force sensors has been proposed in order to know if the electrode is in contact with the tissue during the registration of intracardiac electrograms (EGM). Although threshold criteria on force signals are often used to determine the catheter contact, this may be a limited criterion due to the complexity of the heart dynamics and cardiac vorticity. The present paper is devoted to determining the criteria and force signal profiles that guarantee the contact of the electrode with the tissue. In this study, we analyzed 1391 force signals and their associated EGM recorded during 2 and 8 s, respectively, in 17 patients (82 ± 60 points per patient). We aimed to establish a contact pattern by first visually examining and classifying the signals, according to their likely-contact joint profile and following the suggestions from experts in the doubtful cases. First, we used Principal Component Analysis to scrutinize the force signal dynamics by analyzing the main eigen-directions, first globally and then grouped according to the certainty of their tissue-catheter contact. Second, we used two different linear classifiers (Fisher discriminant and support vector machines) to identify the most relevant components of the previous signal models. We obtained three main types of eigenvectors, namely, pulsatile relevant, non-pulsatile relevant, and irrelevant components. The classifiers reached a moderate to sufficient discrimination capacity (areas under the curve between 0.84 and 0.95 depending on the contact certainty and on the classifier), which allowed us to analyze the relevant properties in the force signals. We conclude that the catheter-tissue contact profiles in force recordings are complex and do not depend only on the signal intensity being above a threshold at a single time instant, but also on time pulsatility and trends. These findings pave the way towards a subsystem which can be included in current intracardiac navigation systems assisted by force contact sensors, and it can provide the clinician with an estimate of the reliability on the tissue-catheter contact in the point-by-point EGM acquisition procedure.

## 1. Introduction

Arrhythmias are prevalent in humans, and globally 2.5% of the population suffers from this disease [1,2,3,4]. This pathology produces a variation in the heart rate, and it can be mostly due to three causes, namely, automatism alteration, triggered activity, and reentry [5,6,7]. The most widely used arrhythmia treatment is cardiac ablation, which consists of determining the diseased area of the heart that originates the arrhythmia, and then canceling the mechanism by using low temperatures or radio frequency [8,9]. The diagnosis and treatment are made in an electrophysiological study, which is an intervention on the patient consisting of the insertion of electrocatheters through the neck or legs veins, and then recording the electrical activity of the heart. This procedure allows the clinician to identify the arrhythmia mechanism and to supply an adequate therapy [10,11]. Systems LocaLisa and Carto${}^{®}$ 3 are widely used examples of this type of equipment, and they basically consist of a positioning subsystem, a set of advanced catheters, and data storage and processing equipment [12,13].

These intracardiac navigation systems are constantly evolving today, and the use of catheters incorporating force sensors is one of the most recent developments. This innovation improves the recording of the heart electrical activity by verifying that the catheter is in contact with the heart surface. In [14], a system of contact force measurements was presented using optical fiber detection technology, which improved the precision of force measurement, and it was benchmarked in terms of the catheter angle and catheter deflection, the fluid irrigation, and the use of a sheath, among other conditions. Another recent study delivered a contact-force control system using a portable catheter controller that can be coupled to any combination of ablation catheters with adjustable sheath and force detection [15]. Neither of the studies reported the use of those systems when applied for clinical data. Other recent works use real patient data, for instance, a comparison was presented in [16] among contact force ablation, manual ablation, and Remote Magnetic Navigation (RMN) ablation, analyzing their safety and efficiency in acute and long-term outcomes. They demonstrated that RMN was superior with regard to acute success, reduction of major complications, and recurrence rate using an intention-to-treat analysis. Another study compared the procedural profiles and outcomes of persistent atrial fibrillation ablation with and without using contact-force sensing catheters. The use of contact-force sensing catheters in this setting was associated with shorter procedures, shorter fluoroscopy time, and reduction in arrhythmia recurrences [17]. Therefore, this has recently been a field in intense technological evolution with respect to the hardware acquisition system and its use.

Studies to date in this setting are based on the use of force thresholds, in such a way that if force sensor registers a reading greater than 5 gr, then the catheter is considered to be well positioned and yielding correct voltage readings [13]. The justification to use this threshold is based on the catheter being surrounded by blood, which usually produces a force lower than 5 gr when interacting with the sensor. However, no study has clearly established that this value can be considered as a consistent and suitable threshold. Moreover, the dynamic nature of the heart functioning presents several challenges when we want to ensure the application of this type of examinations, and one of them is the determination and identification of the time intervals when the catheter is in stable contact with the heart surface to be examined. In a previous study, electroanatomical maps of the electrophysiological examinations were analyzed in a group of patients with arrythmias [13]. These electroanatomical maps were performed with a cardiac navigation system that included a force-sensing catheter. Authors considered healthy tissue when a greater than 5 gr force signal was recorded at the time of the maximum EGM voltage, assumed that its peak-to-peak value was greater than 1.5 mV, and the area was identified as diseased tissue or a scar.

The vorticity produced by the blood and the systole and diastole movements can produce disturbances in the electrical activity measurements, making it difficult to determine diseased areas on the surface of the heart, especially if the criterion of a static threshold is considered as a reference for decision making. In the present work, data exploration was carried out with the purpose of constructing contact models based on the force signal, as a criterion to determine if the catheter is in contact with the tissue. We scrutinize patterns such as pulsatility or trends in the force signals recorded during the electrophysiological examination of a patient, by considering a time window around the EGM maximum peak used for the static force threshold. The Principal Component Analysis (PCA) techniques are used to provide us with a multivariate decomposition of the most characteristic force signal profiles, and linear classification is used to determine their relevance. The patient database used for this purpose consists of 1391 force signals and their corresponding EGM, which were recorded during 2 and 8 s, respectively, from 17 patients (82 ± 60 points per patient) at electrophysiological study.

The outline of the document maintains the following structure. In Section 2, the characteristics of the patient base, the PCA fundamentals, and the used linear classifiers are summarized. Section 3 describes the experimental methods carried out and the obtained results. Section 4 deals with the discussion, future work, and conclusions of our findings.

## 2. Methods

### 2.1. Database and Patients

The patient data used in this work comes from a previous clinical study [13], carried out at the Arrhythmia Unit in University Hospital Virgen de la Arrixaca (Murcia, Spain). That study was approved by the Ethics Committee of the hospital, and recorded in the *clinicaltrials.gov* register of clinical trials under identification number NCT01639365. It involved the acquisition in each patient of a left ventricular voltage map, during sinus rhythm and using the Carto${}^{®}$ 3 navigation system and the Navistar Thermocool catheter (Biosense-Webster, Diamond Bar, CA, United States). The population consisted of 17 patients (8 men, mean age 62, range 38–80 years) with ventricular arrhythmias due to ischemic cardiomyopathy or dilated cardiomyopathy, who underwent ventricular tachycardia ablation by clinical indications, and their clinical description is included in Table 1. For each patient, a voltage map was first generated without accounting for the force signal. Then, the map was corrected by discarding those points with force below threshold, which yielded more compact voltage maps allowing to better identify arrhythmia channels and ablation targets.

The same set of patients and signals was used in this work. The Carto${}^{®}$ 3 System records the electrophysiological examination data in plain text format. Extract, Transformation, and Load techniques were used on these files [18,19], allowing the construction of a database in which anonymized patient identification, case identification, EMG data, and force sensor data were stored. This database facilitated the processing of the acquired signals with a custom-developed computer tool, as well as a subsequent manual data classification, called labeling, where the doctor-issued criteria were followed. This labeling aimed to determine the presence or absence of catheter contact with the tissue not only in terms of the force at a single time instant (EGM peak), but rather taking into account the force signal evolution on a time window around this instant, as well as the simultaneously observed EGM signal. It was evident that sometimes it is complicated to establish the contact with high confidence, hence, the labels allowed two possible levels for the confidence on contact, as considered by the observer. Therefore, four labels were used for this classification. On the one hand, those cases in which the expert observer was confident that there was (not) tissue-catheter contact were given *label 0* (*label 3*). On the other hand, those cases in which there was some reasonable doubt whether there was contact were assigned *label 1* (*label 2*).

**Figure 1:**
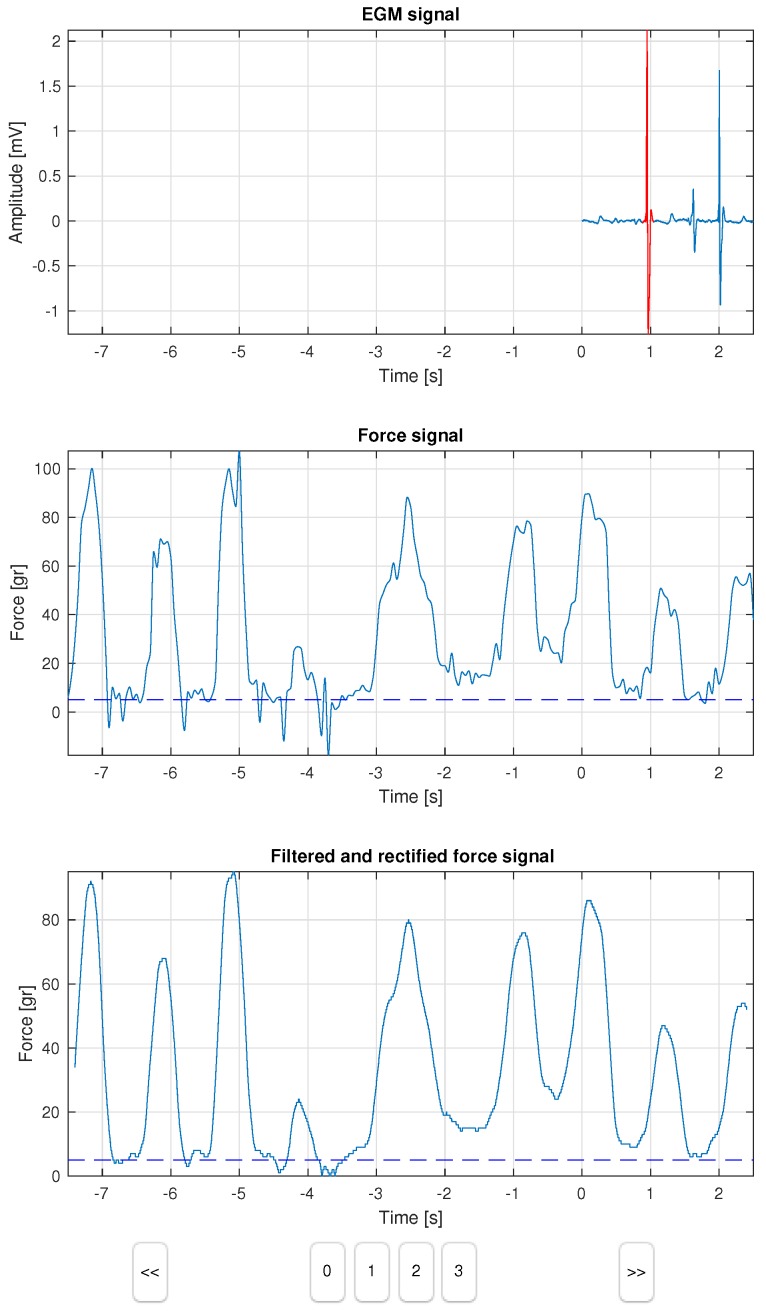
The computer tool dashboard displays the EGM signal, the simultaneously recorded force signal, its filtered and rectified version, and several selection and control buttons.

The developed computer tool consists of a dashboard with 3 panels, as shown in Figure 1. The first panel plots two signals on a shared axis, namely, the 2 s of EGM signal (in blue), and the peak heartbeat selected within a window of ±80 ms (in red). The second panel represents the force sensor signal, which lasts 8 s, and it is synchronized with the EGM signal after 6 s, as seen in the figure. A dashed line represents the 5-gr threshold to be used as a reference by the observer. The third panel provides an additional view of the force signal after filtering and rectification, aiming to better support the labeling in some doubtful cases. Control buttons allowed the label selection, as well as the step forward (>>) and step back (<<) to review the cases. The result of this process was stored in a new label field in the database, which was specific to each case.

### 2.2. PCA Fundamentals

The catheter used for the electrophysiological examination acquires data within a dynamic environment, which exhibit a variety of patterns due to the heartbeat movement and to the blood vorticity. We want to group the different patterns present in a population of force measurements according to their contact labels and label confidence. For this reason, a multivariate technique is required in order to statistically extract the existing patterns and their relevance in that population. For this purpose, in this work we propose to use PCA, a statistical procedure that uses an orthogonal transformation to convert a set of observations of possibly correlated vector observations into a set of linearly uncorrelated vector components. These new vectors and variables are characterized by their mutual uncorrelation. The amount of information conveyed in a component is measured by its variance, which can be used as an indicator to sort them in terms of their relevance, this is, the larger the variance associated to a component, the larger the amount of information in that component.

In statistics, PCA is often used to reduce the dimensionality of a data set, which makes it possible in our case to distinguish among the most relevant dynamic changes in the system, the redundant information, and the noise in the signal [20]. This analysis defines a new coordinate space where the variance is maximized and the correlation among the variables is minimized. In our case, we measure a force signal during 8 s at a given anatomical location of the heart, which is represented by a signal vector $x_{m}\in R^{M}$, where *M* is the total number of signal samples in 8 s. Given a set of training data that contains *N* observation vectors (corresponding to the aggregation of all the cases for every patient in the database), the array $X\in R^{N\times M}$ is constructed with the observed vectors by rows, which represents the original data matrix. The covariance matrix $C_{XX}\in R^{M\times M}$ can be calculated from X in the following way, $C_{XX}=\frac{1}{N-1}X^{⊤}X$. By decomposing this matrix in its eigenvalues and eigenvectors, the correlation structure of the variables is obtained,

(1) $$C_{XX}=V\Lambda V^{⊤},$$

where $\Lambda \in R^{M\times M}$ is a diagonal matrix which contains the non-negative real eigenvalues of $C_{XX}$, this is, $\lambda_{m},$m=1,2,⋯,M, and they are sorted in decreasing order; and matrix $V\in R^{M\times M}$ conveys the sorted eigenvectors in columns. By holding only the *a* eigenvectors (a<M) corresponding to the *a* largest eigenvalues, we can build the so-called reduced principal component matrix $P\in R^{M\times a}$, which projects the observation vector $x\in R^{M}$ into a smaller space $R^{a}$, and it can be denoted by $t^{a}=xP$.

Note that $t^{a}$ represents the optimal projection of x onto the reduced dimensional subspace in terms of the variance. By applying this transformation to the entire data matrix, we obtain $T^{a}=XP$, where $T^{a}\in R^{N\times a}$ represents the new data matrix for the dimension-reduced observation vectors. To obtain an approximation to the original data from this matrix $T^{a}$, we perform the following matrix product, ${\hat{X}}^{a}=T^{a}P^{⊤}$, and then, we can calculate the residual matrix as the difference between the original and the estimated data matrices, as follows, $E=X-{\hat{X}}^{a}$. In many practical applications, a given observation vector is reconstructed with sufficient quality by using only a reduced number *a* of principal components, as seen later in our case.

### 2.3. Fisher and SVM Linear Classifiers

As shown in the experiments, the use of PCA provides us with a set of characteristic patterns on the force signals. Nevertheless, a criterion is further needed to establish the relevance of the reduced set of directions obtained from PCA. For this purpose, we used two linear classifiers, following a similar methodology to the one proposed in [21]. The use of linear classifiers with the PCA projected signals allows us to determine the relevance of each projection direction by scrutinizing their weights. We selected the two linear classifier, namely, the Fisher Linear Discriminant (FLD), as it represents a classic and well-known method, and the linear support vector classifier, which has been successfully used in a number of applications during the last years [22,23].

On the one hand, the FLD is able to find a linear combination of features characterizing two or more sets with classification purposes. The method looks for the projection that maximizes the interclass variance and minimizes the intraclass variance [24,25]. In our case, we work with a set of multivariate observations $t_{n}^{a}$, with n=1,⋯,N, which in general can belong to two possible classes, with mean vectors $m_{0}$ and $m_{1}$ and covariance matrices $\Sigma_{0}$ and $\Sigma_{1}$. The between-class (within-class) dispersion matrix is denoted as $S_{b}$ ($S_{w}$), and they can be obtained by

(2) $$\begin{array}{ccc} S_{b} & = & {\left(m_{1}-m_{0}\right)}^{T}\left(m_{1}+m_{0}\right), \end{array}$$

(3) $$\begin{array}{ccc} S_{w} & = & \sum_{0}+\sum_{1}. \end{array}$$

A linear combination of features $t^{a}w^{T}$ has mean $m_{i}w^{T}$ and variance $w\Sigma_{i}w^{T}$, for i=0,1. Our objective is to maximize the between-class dispersion while minimizing the within-class dispersion, and for this purpose, the discrimination criterion is established as

(4) $$J\left(w\right)=\frac{wS_{b}w^{T}}{wS_{w}w^{T}}$$

It can be shown that the maximum separation is obtained when

(5) $$w^{T}=c{\left(\Sigma_{0}+\Sigma_{1}\right)}^{-1}{\left(m_{1}-m_{0}\right)}^{T}$$

where *c* is a constant. In our case, the weights of Fisher projection vector $w\in R^{a}$ are indicators of the relevance of each of the considered eigenvectors.

On the other hand, the SVM is a supervised learning method that recognizes patterns for data analysis, and it can perform both pattern classification and regression. Vapnik proposed the SVM based on the Statistical Learning Theory, and it is considered one of the most effective classification algorithms [26,27]. In brief, this method uses optimization techniques to determine the classification function, and it consists of minimizing

(6) $$\frac{1}{2}{∥w∥}^{2}+C\sum_{n=1}^{N}\left(\xi_{n}^{*}+\xi_{n}\right)$$

subject to

(7) $$\left\{\begin{array}{c} y_{n}-w^{T}\phi \left(t_{n}^{a}\right)-b\leq \varepsilon +\xi_{n}^{*}\\ w^{T}\phi \left(t_{n}^{a}\right)+b-y_{n}\leq \varepsilon +\xi_{n}\\ \xi_{n}^{*},\xi_{n}\geq 0, \end{array}\right.$$

with n=1,2,…,N, and where w is now the weight vector of the SVM; $\frac{1}{2}{∥w∥}^{2}$ is called the margin and it also accounts for the complexity of the model; *C* is the trade-off parameter between margin and losses; $\xi_{n}^{*}$ and $\xi_{n}$ are the slack variables; $\phi (t^{a})$ is a non-linear transformation that maps the input data to a high dimensional space; and *b* is the interception. Lagrange multipliers $\alpha_{n}$ and $\alpha_{n}^{*}$ are introduced, and then the optimization problem described in the previous equations can be transformed into its dual optimization problem, which consists of maximizing:

(8) $$-\frac{1}{2}\sum_{n,l=1}^{N}\left(\alpha_{n}^{*}-\alpha_{n}\right)\left(\alpha_{l}^{*}-\alpha_{l}\right)y_{n}y_{l}K\left(t_{n}^{a},t_{l}^{a}\right)+\sum_{n=1}^{N}\left(\alpha_{n}^{*}-\alpha_{n}\right)$$

where Mercer’s Theorem has been used to substitute $K\left(t_{n}^{a},t_{l}^{a}\right)=\langle \phi \left(t_{n}^{a}\right),\phi \left(t^{a},l\right)\rangle$, and it is subject to

(9) $$\left\{\begin{array}{c} \sum_{n=1}^{N}\left(\alpha_{n}^{*}-\alpha_{n}\right)y_{n}=0\\ 0\leq \alpha_{n},\alpha_{n}^{*}\leq C \end{array}\right.$$

for n=1,2,⋯,N. The classification function of this SVM is obtained by solving the previous problem, and it can be shown to be

(10) $$\hat{y}=f\left(t^{a}\right)=\sum_{n=1}^{N}\left(\alpha_{n}^{*}-\alpha_{n}\right)y_{n}K\left(t_{n}^{a},t^{a}\right)+b$$

In our case, parameter *C* can be calculated by using out-of-sample techniques, such as cross validation. In addition, several Mercer’s kernels can be used, but here we restrict ourselves to the linear kernel, as follows,

(11) $$K\left(t^{a},u^{a}\right)=\langle t^{a},u^{a}\rangle$$

i.e., it is just the dot product between the two vectors. With this linear kernel, it is possible to calculate the weight vector of the SVM classifier, which is given by

(12) $$w=\sum_{n=1}^{N}y_{n}\left(\alpha_{n}-\alpha_{n}^{*}\right)t_{n}^{a}.$$

Again, the weights of SVM classification vector $w\in R^{a}$ are indicators of the relevance of each of the considered eigenvectors in our problem.

## 3. Experiments and Results

As previously described, our database consisted of 17 patients with a total of 1391 cases (82 ± 60 points per patient). The points in each case were tagged by the expert cardiologists. This database was randomly divided, in such a way that 80% (20%) of the data were in the training (test) set. The PCA was applied to the training group, and the projected vectors resulting from this analysis were used to train the Fisher and SVM classifiers. The trained classifiers were subsequently tested with the test group. Each process is specifically described below.

### 3.1. Results with the Conventional Force Threshold

With respect to the labels established by the experts on the catheter-tissue contact, a nomenclature was used to establish the vector groups according to the different confidence on the labels. On the one hand, cases with *label 0* were included in the *high-confidence contact* group, whereas cases with *label 3* were in the *high-confidence non-contact* group. On the other hand, cases with label either 0 or 1 were included in the *likely contact* group, whereas cases with *labels 2* or *3* were in the *likely non-contact* group. Note that the first group accounts for those cases that were more clearly identified in terms of contact or not by the expert, whereas the second group also includes those doubtful cases in the analysis.

**Figure 2:**
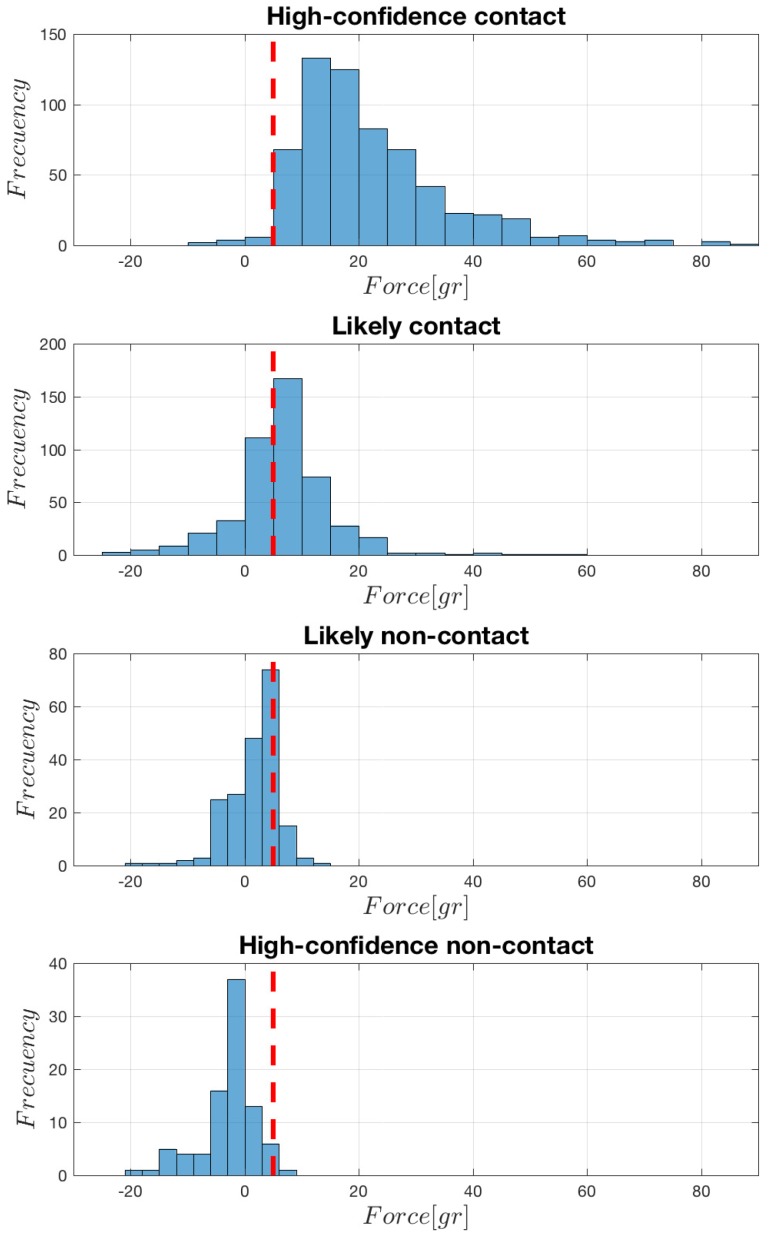
Histograms of the force signal values at the time of the EGM peak, according to the conventional criterion. The 5-gr threshold is depicted (vertical red line).

The histogram representation of the force signals as recorded at the peak time of each corresponding EGM is shown in Figure 2. When analyzing each of the groups, it can be seen that the high-confidence contact group has a range about −10 gr to 80 gr of force applied to the sensor, in most cases above the 5-gr threshold. In the case of the likely contact group, it has a range between −25 gr to 60 gr, with the histogram bin around the threshold being about the distribution mode. In the case of the likely non-contact group, the force values present a range from −25 gr to 15 gr, and most of the signal samples are below the threshold. Finally, in the case of the contact high-confidence non-contact group, the force sample distributions are quite similar, though with a general amplitude decrease in the second one. Note the presence of negative tails in all the groups, specially (but not only) in the non-contact ones. Note also that discriminating between contact and non-contact by using the threshold can produce some number of misclassifications, as an overlap zone exists among groups, and the most likely error to happen is to establish that a point has no contact when it really represents a tissue-catheter position.

### 3.2. PCA on Force Signals

**Figure 3:**
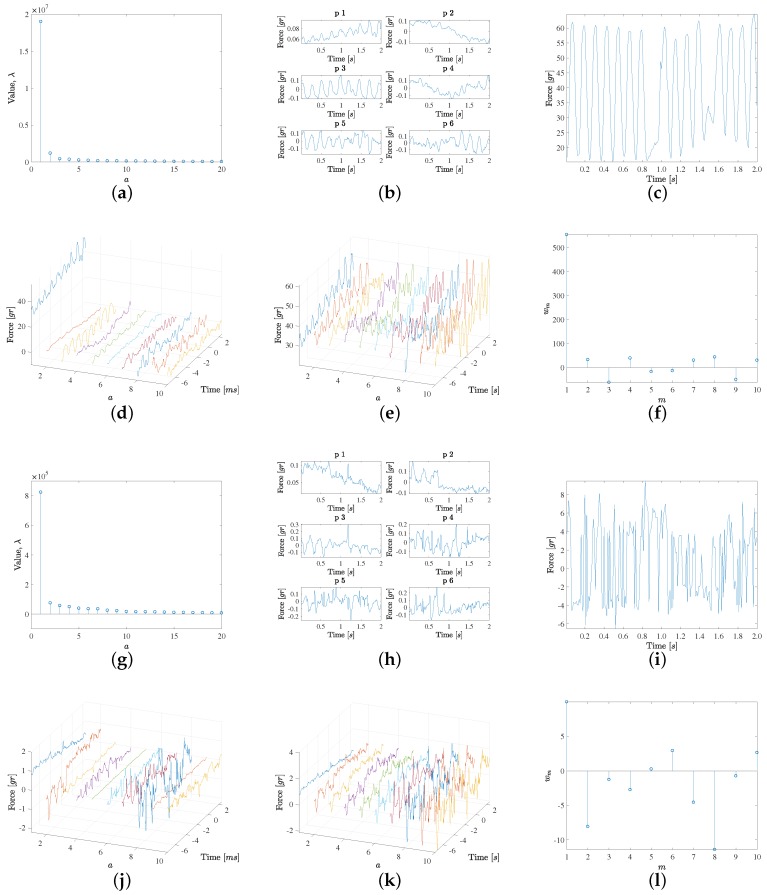
PCA results for the high-confidence contact (**a**–**f**) and non-contact (**g**–**l**) groups, separately: (**a**,**g**) First 20 eigenvalues; (**b**,**h**) Signal patterns for the first six eigenvectors of the decomposition; (**c**,**i**) Examples of signals with clearly stable and loose contact; (**d**,**j**) Contributions of the first 10 eigendirections weighted by the example signal projection on them; (**e**,**k**) The same for the accumulated contributions; (**f**,**l**) Weights of the projected example signal on those first 10 eigenvectors.

The PCA was used to scrutinize the data patterns of the force signals, first for each of the labels separately, and then in the two described sets of grouped labels. The following three figures that are next presented are related to the PCA of the different labels and label groups, and they follow the same structure and use six panels for each analysis, see Figure 3. Panel (a) depicts the first 20 eigenvalues, hence yielding an idea of the relevance of their corresponding eigendirections, and Panel (b) represents the first six eigenvectors in order to be able to inspect their waveforms and to establish the characteristic signal patterns for the analyzed groups. Panel (c) shows a representative force signal example in each group, and then, the contributions of up to 10 eigendirections, as weighted by the projection of the example signal on them, are represented separately (Panel (d)) and accumulated (Panel (e)), in such a way that the convergence to a quality estimation (reduced residual) of the example signal can be readily scrutinized. Finally, Panel (f) shows the weights of the projected example signal on the first 10 eigenvectors, to give an idea of the relevance of each of the projected weights in that example.

Figure 3 shows the results obtained by analyzing the high-confidence contact (non-contact) groups from (a) to (f) (from (g) to (l)). The high-confidence contact group exhibits the following characteristics. On the one hand, the first eigenvalue is clearly the most relevant one and it is associated both to visible pulsatility as well as an increasing trend. This trend is consistent with the observed increased force values during the last two seconds when the EGM are recorded at each point. On the other hand, the second eigenvalue shows a decreasing trend, again consistent with those cases when the local average force is reduced at the end of the force recording period. Note that these patterns can be present either as plotted or with negative sign, according to the sign given by the projection of each specific force case. Additionally, the pulsatility is mostly related with the average cardiac rhythm cycle, but there are changes in the pulsatile force waveforms for each specific case, and these differences are provided by eigenvectors 3 and 5, which are associated to a moderately large eigenvector, but this is enough to contribute with the relevant waveform information in this set of cases. It can be seen too that eigenvector 4 provides an additional trend pattern (high, low, high again), whereas eigenvector 6 is a mixed one, given by noisy activity followed by a pulsatile pattern in the end of the signal, i.e., in the time interval where the EGM has been simultaneously recorded. Later, the following eigendirections seem to provide few relevant contributions to the signal patterns. In order to verify this, a signal example was chosen where the contact was clear and stable, in terms of pulsatility and average value, as seen in Figure 3c. In this case, Panel (d) shows the eigendirections weighted by the example projection weights in Panel (f). While weights 7 to 10 in the weight vector obtained for a=10 seem to be numerically relevant, it can be seen in Panel (e) that the accumulated signal reconstruction is very close to the original one with the first six components; hence, this group exhibits high signal-to-noise ratio.

Figure 3 also shows in Panels (g) to (l) the results for the high-confidence non-contact group, which has the following characteristics. The first eigenvalue is markedly smaller than in the case of the high-confidence contact group, but still it is markedly larger than the other eigenvalues in its group. In this case, this first eigendirection corresponds to a decreasing trend. It can be seen that the six first eigendirections have some moderate pattern of pulsatility, but there is no specific pulsatile direction, and instead it is at most mixed among random patterns. The example scrutinized in this group, at Panel (i), is again built with enough quality with about the first eight eigendirections. Note that in this case, the contribution of the first eigendirection is markedly smaller, as this example has a small average value.

**Figure 4:**
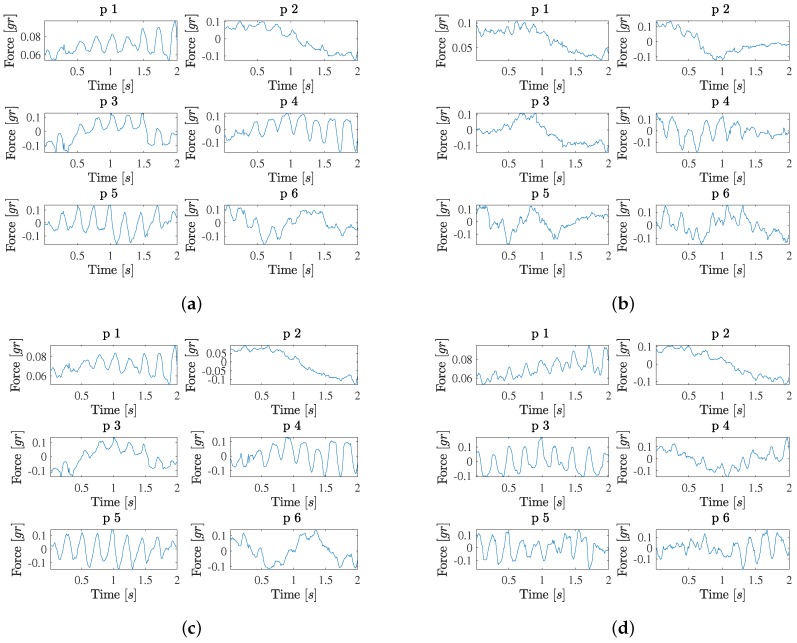
PCA results and signal patterns for the first 6 eigenvectors of the decomposition in likely contact (**a**), likely non-contact (**b**), likely (**c**), and high-confidence (**d**) groups, using all cases and both contact and non-contact groups, separately.

Figure 4 shows the results obtained when analyzing the likely-contact (likely non-contact) groups separately, in Panels (a) (Panel (b)). The eigenvalue profile in the contact group is similar to the high-confidence contact group, but now the most relevant profiles in Panel (b) often include mixed pulsatility and trends (eigendirections 1, 2, 3, 4, and 5). In addition, a new type of pattern can be seen, which consists of pulsatility that is modulated with its amplitude increasing to the end of the time interval (eigendirections 1 and 4). Again, the signal is estimated with acceptable quality by using 8 eigenvectors. A moderate signal-to-noise can be observed now in the force signals in this group. The eigenvalue profile in the likely non-contact group is also similar to the high-confidence non-contact group, though the first eigenvalue (corresponding to the group average) is in this case larger. Now, the first relevant eigendirections also include mixed patterns of trends with some presence of pulsatility (e.g., as in eigendirections 3 and 4).

After analyzing the patterns of the different contact and non-contact groups separately, we examined the patters of the complete database, as seen in Figure 4. On the one hand, the likely-contact group (all cases together) shows an eigenvalue profile which is similar to the previous ones (and more similar to the contact subgroups). Current relevant patters in this complete set include a mixture of purely pulsatile waveforms (eigendirection 5), pulsatility with increasing amplitude (eigendirections 1 and 4), trends with some pulsatility (eigendirections 3 and 6), and just trends (eigendirection 2). On the other hand, in the high-confidence contact group (cases with *labels 0* and *3*), eigendirections 1 and 2 are very similar to the ones in the complete dataset, and the patterns include purely pulsatile waveforms (eigendirections 3 and 5), pulsatility with increasing amplitude (eigendirection 1), trends with small pulsatility or pulsatility at the end of the signal (eigendirections 2, 4, and 6). Overall, we can check in all the examples that 8 to 10 components yield acceptable quality in the force signal reconstruction.

### 3.3. Classification Using PCA Weights

The weights from the projection of each force signal on the eigenvectors of different groups were shown for some examples in the previous section. These weights can be used to analyze which are the most relevant eigendirections and also their corresponding patterns, when analyzed in the complete data set.

**Figure 5:**
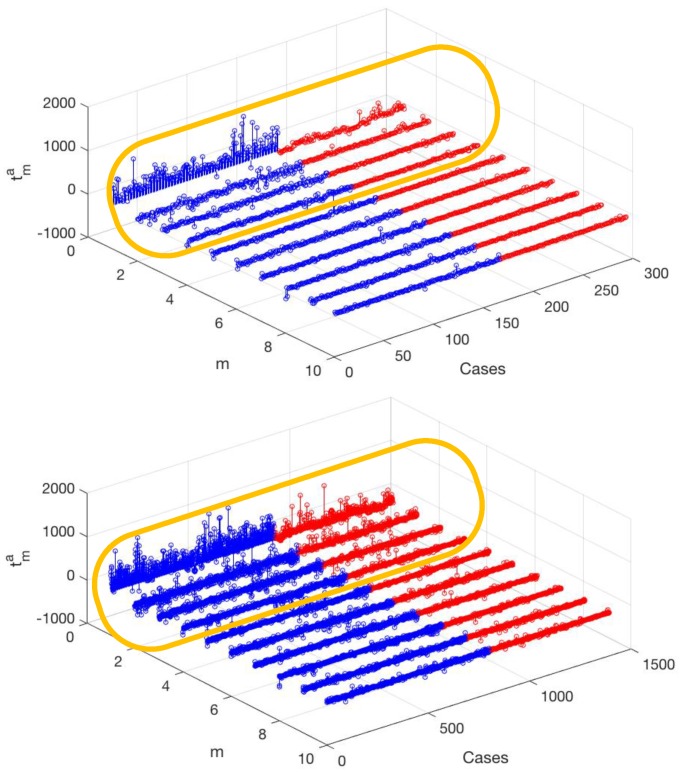
Representation of the weights given by the projection of each force signal case on the first 10 eigenvectors, for the contact (blue) and non-contact (red) cases, for a=10, in the high-confidence (**up**) groups and on the complete database (**down**). The oval emphasizes that the differences are more visually patent on the first four weights, in such a way that larger variance can be observed on them. Nevertheless, the other components also exhibit statistical differences, which can be less present to the naked eye, but can be exploited from statistical data processing techniques.

This has been addressed in Figure 5 (upper panel), where the projection weights $t_{m}^{s}$ are represented for a=10 and both in the high-confidence contact and non-contact (blue and red) data matrices. It can be seen that the weight of the first eigenvector is usually positive and notably larger in the contact than in the non-contact cases, nevertheless, the greatest amount of information is concentrated on it. The other weights (2 to 10) show a similar structure among them, given in the high-confidence contact cases by usually small weights together with a few greater weights, though the feature number of the prominent weights differ in each case. In addition, in general they are visibly smaller and distributed around zero in the high-confidence non-contact cases. With respect to the likely-contact cases (lower panel), the first weight is again prominent in the contact cases, but it is also noticeably higher than in the non-contact cases. The other weights (2 to 10) consist both on small values together with some larger positive and negative weights, but the number of larger weights seems to be higher in the contact cases. Accordingly, differences exist in the weights of contact and non-contact cases, but they cannot be immediately used quantitatively in order to discriminate if the catheter is in contact or not. Moreover, it is desirable to quantify how relevant each of the eigendirections are in the database.

For this purpose, we used the projected vectors of the signal force cases (again with a=10) to build several linear classifiers, which were trained and tested with 80% and 20% of the database cases, respectively. Note that only the training samples were used to perform the PCA projection matrices. This procedure was repeated 10 times, which allowed us to scrutinize the distribution of the estimations for each classifier weight.

The classification results for this experiment are shown in Table 2, where the Area Under the Curve (AUC), is shown for the two classifiers applied to the different contact groups. Note the AUC can be seen as a merit figure for the classifiers, in such a way that the closer to one, the lower overlap between the two scrutinized classes. The likely-contact vs. likely non-contact classifiers exhibit greater overlap between classes, although 0.85 is a sufficient AUC value to indicate that the features given by the PCA projections in this case contain relevant information about the classification. The high-confidence contact vs. high-confidence non-contact classifiers yielded AUC average of 0.95, which represents a very good quality classifier and the features used for this classification can also be scrutinized to identify the most relevant patterns. As shown in the table, the AUC medians are similar to the means and the standard deviations are small in all the classifiers.

**Figure 6:**
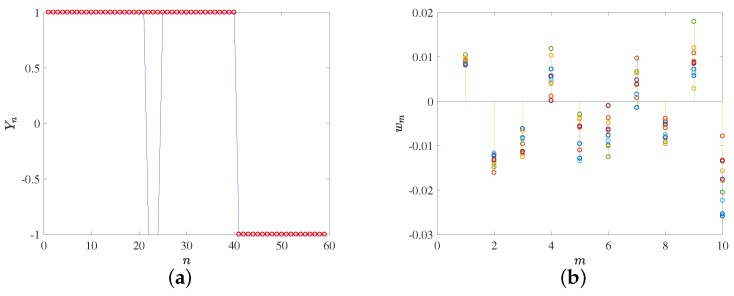

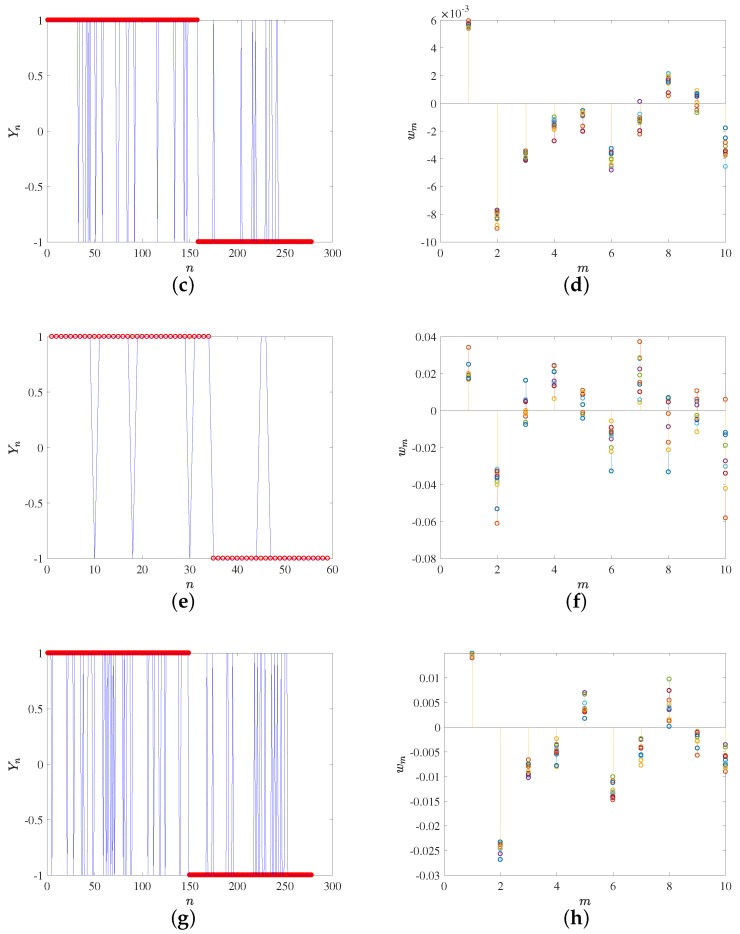
Classification results for FLD (**a**–**d**) and SVM (**e**–**h**), for high-confidence contact (**a**,**b** and **e**,**f**) and likely contact (**c**,**d** and **g**,**h**) groups. Left panels show target classification (red points) and machine-provided classification (in blue). Right panels depict the vector weights for the classifiers and for the 10 repetitions.

Further details on the classification performance can be seen in Figure 6, where the left panels show the actual group and the estimated group for each classifier. Classification errors are more patent in the low-AUC classifiers, at the likely contact vs. non-contact groups, and these errors in general are similarly distributed between classes. However, in the high-AUC classifiers, the scarce errors only (SVM) and mostly (FLD) correspond to actual contact cases which have been misclassified as non-contact ones.

After checking that the classifiers provide enough performance in terms of the AUC, it is possible to study the relevance and significance of the weights in each classifier and to interpret the clinical meaning of the trained machines. Recall that each classification weight is associated to a given projection weight, so that by looking at its corresponding eigenvector it is possible to determine the patterns that are actually relevant to the classification. In Figure 6 (right panels), the weights for the different classifiers are represented. In the likely contact vs. likely non-contact group classifiers, we can see that classifier weights 3 to 10 are in a rank which either overlaps zero or is very close to it, at least in one of the two classifiers. Therefore, it can be inferred that weights 1 and 2 are the most relevant to distinguish between these clearly differentiated classes, which corresponds to eigenvectors 1 and 2 in Figure 4a,b, and hence to a pulsatile with increasing amplitude pattern and to a biphasic trend pattern. These patters convey enough information to distinguish between contact and non-contact groups. Note that classification weight 1 is always positive, which is consistent with the positive and larger mean in class y=1 in the classifier (contact), and also, classification weight 2 is negative and multiplying a decreasing trend, which is consistent with an increased trend value in the end of the contact cases. In the high-confidence group classifiers, classifier weights 1 and 2 exhibit a similar trend to the previous case, and they are very similar, which is consistent with the similarity found in Figure 4c,d on the two first eigendirections for both contact and non-contact groups. In this case, several other weights become relevant in both classifiers, namely, classifier weights 3, 4, 6, and 10. The patterns in these eigendirections allow a better description of the details required in the easier cases.

## 4. Discussion and Conclusions

In this work, the determination of the catheter-tissue contact in electrophysiological studies for arrhythmia therapy has been analyzed in terms of the force signals recorded in force sensors at the EGM recording catheter. The conventional criterion of using a single-sample threshold of 5-gr as measured at the EGM peak time has been shown to be limited for the clinical practice. A previous study from our group aimed to use simple statistical features, such as the correlation coefficient, in order to scrutinize the improvement of tissue-catheter contact, but these simple features were not enough to give an operative system [28]. The use of PCA has allowed us to establish the relevant patterns that can be found in this type of signal, and the use of linear classifiers has further allowed the establishment of the suitability of these signals to identify the existence of contact or non-contact in an acceptable number of cases.

Several limitations to the presented study can be identified. The labeling of contact vs. non-contact was done by experts, but still it can sometimes be subjective. An objective index would undoubtedly improve the performance and quality of the proposed system. Nevertheless, the proposed methodology would be readily and similarly used in that case. The use of linear classifiers could be improved by nonlinear classifiers in terms of system accuracy; nevertheless, here we restricted ourselves to the first ones because they allow to scrutinize the relevant patterns for the classification, and hence to provide clinical understanding of the problem at hand and of the proposed solution. Nevertheless, both the signal processing procedures and the implementation of the tuned machine learning classifiers require a very moderate and acceptable computational burden.

We can conclude that the proposed system can be used to determine the presence of tissue-catheter contact during arrhythmia procedures, which would improve the quality of the maps elaborated by current intracardiac navigation systems, and in general the improvement of the functionality provided by current force sensors at EGM catheters. The proposed method could be used as a subsystem for current cardiac navigation systems supporting the clinical ablation procedures in two ways. On the one hand, it can be used on-line while recording the EGM and creating the maps, so that the clinician is advised of the likely good quality of the tissue-catheter contact, hence only those points which are clearly in contact are accepted. This represents a similar concept to the use of thresholds in current systems, but the use of signal processing profiles should improve this quality. On the other hand, they could be used off-line, after taking the patient measurements, in order to discard those points which were less-likely high-quality contact, and hence improving the final map. In both cases, force-signal profiles should be taken into account and would be advantageous to the clinical procedure.

## Figures and Tables

**Table 1:** Demographic and clinical characteristics.

Patient	ICD	Heart Disease	Number of Infarctions	Location	LVEF	LVEDD	LVESD	Septum	Spontaneous VT Morphology	Induced VT Morphology
1	Yes	Ischemic	1	Inferior	40	60	52	11	2 ECG ICD	No
2	Yes	Ischemic	1	Inferior	45	44	31	13	No	1 RBBB SUP
3	Yes	Ischemic	1	Anteroapical	25	52	36	13	1 ECG ICD	No
4	Yes	Ischemic	2	Anterior/inferior	31	78	59	6	2 ECG ICD	No
5	No	Ischemic	1	Inferior	51	52	36	13	1 RBBB SUP	1 RBBB SUP
6	No	Ischemic	2	Inferior/septal	43	54	48	10	1 VES: RBBB SUP	No
7	No	Dilated	0	NA	20	67	63	11	1 LBBB SUP	1 LBBB SUP
8	Yes	Ischemic	0	NA	20	60	40	9	1 ECG ICD	No
9	No	Dilated	0	NA	20	59	54	8	4 VES: RBBB INF	No
10	No	Ischemic	1	Septal	28	51	35	9	1 RBBB SUP	1 RBBB SUP
11	No	Ischemic	1	Posteroinferior	45	50	37	12	RBBB SUP	2 polymorphic
12	Yes	Ischemic	1	Anterolateral	37	59	47	10	ECG ICD	LBBB INF
13	Yes	Ischemic	1	Anterior	30	56	47	11	ECG ICD	No
14	No	NC	0	NA	62	53	42	12	LBBB INF	RBBB INF
15	No	Ischemic	1	Inferolateral	50	NA	NA	NA	LBBB SUP	No
16	No	Ischemic	1	Anteroapical	32	NA	NA	16	RBBB SUP	No
17	No	Ischemic	1	Inferolateral	45	NA	NA	NA	RBBB SUP	RBBB SUP

RBBB, Right Bundle Branch Block; LBBB, Left Bundle Branch Block; ICD, Implantable Cardioverter Defibrillator; ECG, Electrocardiogram; LVEF, Left; Ventricular Ejection Fraction; LVESD, Left Ventricle End-Systolic Diameter; INF, Inferior Frontal Axis; NA, Not Applicable; SUP, Superior Frontal Axis; VT, Ventricular Tachycardia; VES, Ventricular Extrasystole; NC, Non Compaction Cardiomyopathy.

**Table 2:** Results of FLD and SVM classifiers in terms of AUC, where mn, md, and sd denote the mean, median, and standard deviation through all the cross-validation runs.

Classifier	Classified Groups	mn	md	ss
*FLD*	*High-confidence contact vs High-confidence non-contact*	0.95	0.96	0.02
*FLD*	*Likely contact vs Likely non-contact*	0.84	0.83	0.02
*SVM*	*High-confidence contact vs High-confidence non-contact*	0.94	0.94	0.02
*SVM*	*Likely contact vs Likely non-contact*	0.85	0.85	0.01

## References

[1] Al-Khatib S.M., Stevenson W.G., Ackerman M.J., Bryant W.J., Callans D.J., Curtis A.B., Deal B.J., Dickfeld T., Field M.E., Fonarow G.C. (2017). 2017 AHA/ACC/HRS Guideline for Management of Patients With Ventricular Arrhythmias and the Prevention of Sudden Cardiac Death: Executive Summary. Circulation.

[2] Kirchhof P., Benussi S., Kotecha D., Ahlsson A., Atar D., Casadei B., Castella M., Diener H.C., Heidbuchel H., Hendriks J. (2016). 2016 ESC Guidelines for the management of atrial fibrillation developed in collaboration with EACTS. EP Eur..

[3] Yang H., Kuijpers J., de Groot J., Konings T., van Dijk A., Sieswerda G., Post M., Mulder B., Bouma B. (2017). Impact of atrial arrhythmias on outcome in adults with congenital heart disease. Int. J. Cardiol..

[4] Limongelli G., Sarubbi B. (2017). Atrial arrhythmias in adults with congenital heart disease. Listening to your heart sound can save your life. Int. J. Cardiol..

[5] Peters N. (2000). Arrhythmogenic mechanisms: Automaticity, triggered activity, and reentry. Cardiac Electrophysiology: From Cell to Bedside.

[6] Li H., Yuan D., Wang Y., Cui D., Cao L. (2016). Arrhythmia Classification Based on Multi-Domain Feature Extraction for an ECG Recognition System. Sensors.

[7] García Iglesias D., Roqueñi Gutiérrez N., De Cos F.J., Calvo D. (2018). Analysis of the High-Frequency Content in Human QRS Complexes by the Continuous Wavelet Transform: An Automatized Analysis for the Prediction of Sudden Cardiac Death. Sensors.

[8] Álvarez M., Merino J.L. (2002). Spanish Registry on Catheter Ablation. 1st Official Report of the Working Group on Electrophysiology and Arrhythmias of the Spanish Society of Cardiology (Year 2001). Revista Española de Cardiología.

[9] Garg J., Shah N., Krishnamoorthy P., Mehta K., Bozorgnia B., Boyle N., Freudenberger R., Natale A. (2017). Catheter ablation of accessory pathway: 14-year trends in utilization and complications in adults in the United States. Int. J. Cardiol..

[10] Helguera M.E., De Elizalde G., Maid G., Corrado G., Cagide A., Doval H., Krauss J., Vulcano N., Belziti C., Estrada J.L.N. (2003). Ablación por radiofrecuencia para el tratamiento de las arritmias cardíacas en 500 pacientes consecutivos. Rev. Argent. Cardiol..

[11] Hu S., Wei H., Chen Y., Tan J. (2012). A Real-Time Cardiac Arrhythmia Classification System with Wearable Sensor Networks. Sensors.

[12] Abello M., Merino J.L., Peinado R., Gnoatto M., Arias M.A., Vasserot M.G., Sobrino J.A. (2004). Ventricular Tachycardia Ablation Guided by LocaLisa System in Patients With Structural Heart Disease. Revista Española de Cardiología.

[13] Sánchez Muñoz J.J., Peñafiel Verdú P., Martínez Sánchez J., Salar Alcaraz M., Valdés Chavarri M., García Alberola A. (2015). Usefulness of the Contact Force Sensing Catheter to Assess the Areas of Myocardial Scar in Patients With Ventricular Tachycardia. Revista Española de Cardiología.

[14] Bourier F., Gianni C., Dare M., Deisenhofer I., Hessling G., Reents T., Mohanty S., Trivedi C., Natale A., Al-Ahmad A. (2017). Fiberoptic Contact-Force Sensing Electrophysiological Catheters: How Precise Is the Technology?. J. Cardiovasc. Electrophysiol..

[15] Gelman D., Skanes A., Tavallaei M., Drangova M. (2016). Design and Evaluation of a Catheter Contact-Force Controller for Cardiac Ablation Therapy. IEEE Trans. Biomed. Eng..

[16] Hendriks A., Akca F., Dabiri Abkenari L., Khan M., Bhagwandien R., Yap S.C., Wijchers S., Szili-Torok T. (2015). Safety and clinical outcome of catheter ablation of ventricular arrhythmias using contact force sensing: Consecutive case series. J. Cardiovasc. Electrophysiol..

[17] Hussein A., Barakat A., Saliba W., Tarakji K., Bassiouny M., Baranowski B., Tchou P., Bhargava M., Dresing T., Callahan T. (2017). Persistent Atrial Fibrillation Ablation With or Without Contact Force Sensing. J. Cardiovasc. Electrophysiol..

[18] Tang Y., Mackey I., Su J. (2018). Querying Workflow Logs. Information.

[19] Madhikermi M., Buda A., Dave B., Främling K. Data Model Logger—Data Discovery for Extract- Transform-Load. Proceedings of the 2017 IEEE 19th International Conference on High Performance Computing and Communications; IEEE 15th International Conference on Smart City; IEEE 3rd International Conference on Data Science and Systems (HPCC/SmartCity/DSS).

[20] Quiroga J., Mujica L., Villamizar R., Ruiz M., Camacho J. (2017). PCA Based Stress Monitoring of Cylindrical Specimens Using PZTs and Guided Waves. Sensors.

[21] Gorostiaga A., Rojo-Álvarez J.L. (2016). On the use of conventional and statistical-learning techniques for the analysis of PISA results in Spain. Neurocomputing.

[22] Vapnik V.N. (1998). Statistical Learning Theory.

[23] Rojo-Álvarez J., Martínez-Ramón M., Camps-Valls G., Muñoz-Marí J. (2018). Digital Signal Processing with Kernel Methods.

[24] Fisher A.R. (1923). The Mathematical Theory of Probabilities.

[25] Fisher A.R. (1952). Contributions to Mathematical Statistics.

[26] Men H., Fu S., Yang J., Cheng M., Shi Y., Liu J. (2018). Comparison of SVM, RF and ELM on an Electronic Nose for the Intelligent Evaluation of Paraffin Samples. Sensors.

[27] Cortes C., Vapnik V. (1995). Support-vector networks. Mach. Learn..

[28] Rivas D., Huerta M., Sanroman-Junquera M., Sanchez-Munoz J., Garcia-Alberola A., Rojo-Àlvarez J. (2016). A quantitative analysis on the intracardiac electrogram contact during ventricular tachycardia ablation. Comput. Cardiol..

